# ZrB_2_–Copper–Graphite Composite for Electric Brushes: Positive Effect of ZrB_2_ Addition on Composite Properties

**DOI:** 10.3390/ma17122980

**Published:** 2024-06-18

**Authors:** Yuqiang Feng, Feng Ding, Shuxin Wang, Chengnan Zhu

**Affiliations:** 1 School of Intelligent Manufacturing, Shanghai Zhongqiao Vocational and Technical University, Shanghai 201514, China; fengyq@shzq.edu.cn (Y.F.);; 2 School of Materials Science and Engineering, Shanghai University, Shanghai 200444, China

**Keywords:** ZrB_2_, electric brushes, hardness, bending strength, wear resistance

## Abstract

A ZrB_2_–copper–graphite composite was produced through powder metallurgy and was tested as a new electric brush material. The aim of this paper was to study the effect of ZrB_2_ addition on the composite’s properties. Besides its physical properties such as density and resistivity, its mechanical properties, such as hardness, bending strength and wear resistance, were studied. A scanning electron microscope (SEM) was used to study the morphology of the wear surface, and a configured energy-dispersive spectrometer (EDS) was used to research the chemical composition of the samples. The results showed that, with the addition of ZrB_2_, the composite’s properties such as density, resistivity, hardness, and bending strength improved significantly. Compared with samples without ZrB_2_, samples with the addition of 4% ZrB_2_ achieved a hardness of 87.5 HRA, which was improved by 45.8%, and a bending strength of 53.1 MPa, which was increased by nearly 50.0%. Composites with 1% content of ZrB_2_ showed the best wear resistance under non-conductive friction; however, under conductive friction, composites with 4% content of ZrB_2_ showed better wear resistance.

## 1. Introduction

Copper alloys are widely used in fields such as motors, electronics, machinery, aviation, and aerospace due to their high electrical and thermal conductivity, good corrosion resistance, and ease of production [1,2,3]. However, their high manufacturing cost and energy consumption during production limit their extensive applications in certain fields, especially for small-batch production and customized sample production. Copper matrix composites are recognized as suitable materials in these applications due to their enhanced strength, dimensional stability, wear resistance, and flexible production. Hexagonal ZrB_2_ belongs to the group of ultra-high temperature ceramics with a melting temperature of 3246 °C and excellent mechanical properties because of its strong covalent and ionic–covalent bonds between atoms [4,5]. Prasad et al. [6] studied the influence of ZrB_2_ on the wear and mechanical properties of aluminum alloy and found an increase in tensile strength of 18.8%, increase in hardness of 64.2%, and significant reinforcement in wear resistance. Dinaharan et al. [7] found an increase in tensile strength and hardness with increasing content of ZrB_2_ in aluminum alloy. Fiantok et al. [8] researched a ZrB_2_ thin film alloyed with Ag, and their conclusions indicated a significant improvement in ductility and tribological properties but at the cost of reduced hardness. Ding et al. [9] investigated the addition of ZrB_2_ into Mo-9Si-8B alloys and found that doping 2.5 wt.% ZrB_2_ could obviously increase the fracture toughness of the alloy and achieve the effect of reducing mass loss by 80.3%. Guan et al. [10] prepared ZrB_2_ nanoparticles in an Al matrix and obtained a significant shear stress improvement (from 255 MPa to 491 MPa); meanwhile, a dislocation-strengthening effect caused by dispersed ZrB_2_ particles was observed.

In a typical structure of direct-current generators, copper–graphite composites have been used as brush materials in such generators for many years [11,12]. However, with the growing demand for high-performance generators, traditional brush materials are facing challenges such as lower wear resistance under high temperatures and short service life [13,14]. Moustafa et al. [15] and Hu et al. [16] investigated the possibility to produce copper-coated graphite composites to increase surface wear resistance. Although wear resistance was slightly improved, the cost was high. Yakut et al. [17] added ZrB_2_ into spherical graphite cast iron to investigate the effect on its mechanical properties and came to the conclusion that samples with the addition of 0.455% ZrB_2_ showed the highest hardness (243 HB) and compressive strength (1438 MPa). Sulima et al. [18] studied ZrB_2_–copper matrix composites produced by ball milling and spark plasma sintering and found a strong decrease in the electrical conductivity of composites containing 5% and 10 wt.% ZrB_2_, as well as in those containing larger amounts, due to the partial diffusion of boron into the copper matrix, although the hardness and Young’s modules of the samples were significantly improved. Zhao et al. [19] prepared ZrB_2_-SiC- reinforced copper matrix composite coatings by laser cladding and found that the microhardness of the composite was about 5.6 times that of the copper matrix.

When the literature was researched, it was observed that there were not sufficient studies on adding both ZrB_2_ and graphite together into the copper matrix. In this paper, ZrB_2_ and graphite were mixed together in a certain ratio into the copper matrix to study their effect on the properties of the composite. The ZrB_2_–copper–graphite composite was produced through hot-press sintering. Besides its mechanical properties, such as hardness, bending strength, and wear resistance, its physical properties, such as density and resistivity, were investigated as well. In order to better simulate the working conditions of electric brushes, a piece of conductive friction test equipment was designed to compare the composite’s performance with that under non-conductive friction. The aim of this manuscript is to study the effect of adding ZrB_2_ on the properties of copper–graphite matrixes.

## 2. Materials and Methods

### 2.1. Preparation of Materials and Samples

The materials used in this experiment are as follows: (a) nano-ZrB_2_ powder with an average radius of 50 nm (99.9% purity, Hefei Kaier Nanotechnology Co., Ltd., Hefei, China); (b) copper powder with an average radius of 50 μm (99.9% purity, Beijing Youyan Powder Co., Ltd., Beijing, China), which was electro-generated; (c) graphite powder with an average radius of 50 μm (99.9% purity, Shandong Nanshu Graphite Mine Co., Ltd., Qingdao, China). The powders were then mixed into 5 groups according to the ratios shown in Table 1 to produce ZrB_2_–copper–graphite composites.

**Figure 1:**
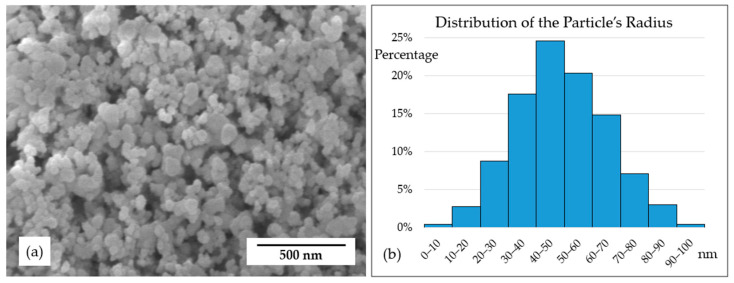
Characterization of ZrB_2_ nanoparticles: (**a**) morphology under SEM; (**b**) radius distribution of the particles.

Figure 1 displays the morphology of ZrB_2_ nanoparticles under SEM. ZrB_2_ particles with an average radius of 50 nm appear in a cluster instead of as single units due to the electrostatic adsorption effect of nanoscale particles.

The mixed powders were then suppressed under a pressure of 300 MPa with a holding time of 20 s. A sample mold with a dimension size of 32 mm × 8 mm × 5 mm was used for suppressing. After suppressing, samples were sintered under vacuum conditions by hot-press sintering under 35 MPa at 860–890 °C for 2 h. According to the literature, ZrB_2_ exhibits a high degree of consolidation when the temperature is above 850 °C [18].

### 2.2. Experimental Procedure

The microstructure of the composites was observed through an optical microscope (GX51, Olympus, Tokyo, Japan) and scanning electron microscope (SEM, Zeiss Gemini 300, Oberkochen, Germany).

The density of the sintered samples was measured by the volumetric method with a high-accuracy electronic balance (XPR204S, Mettler-Toledo, Zurich, Switzerland), which was used to measure the mass loss of samples during the friction and wear test as well. A resistivity-measuring instrument (ST2811, Jing Ge Electronics Co., Ltd., Suzhou, China) was used to measure the resistivity of the samples with voltammetry according to the national standard JB/T 8133.13-2013 [20]. A Rockwell hardness tester (HR150-A, Hua Yin Testing Machine Technology Co., Ltd., Laizhou, China) was employed in this experiment with a load of 60 kg and a holding time of 20 s. A three-point bending test was conducted with a loading rate of 0.05 mm/min and a 10 mm span to acquire the bending strength of the samples using a universal testing machine (AG-Xplus, Shimadzu, Kyoto, Japan). For each sample group, at least three specimens were tested and one of them was reserved for backup.

**Figure 2:**
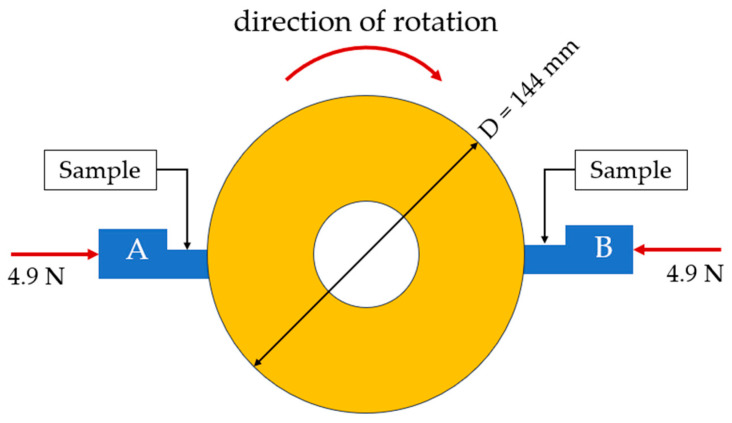
Schematic diagram of the friction and wear testing device.

**Figure 3:**
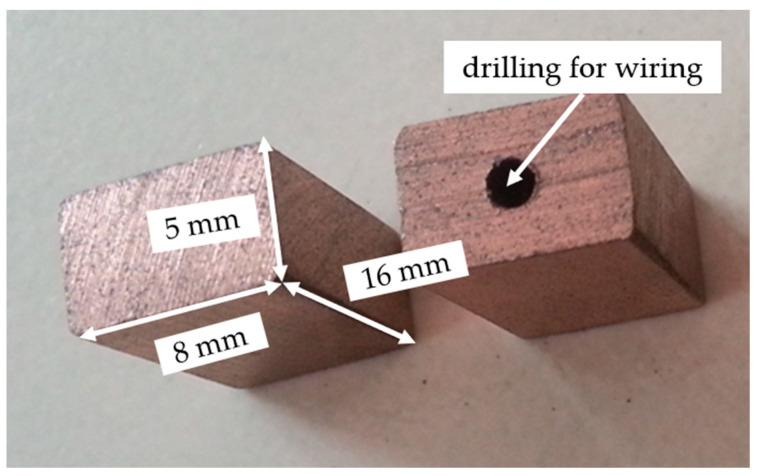
Samples of ZrB_2_–copper–graphite composite after sintering and cutting.

Friction and wear experiments were carried out on a self-made sliding electric contact device, which was designed according to the national standard JB/T 8155-2017 [21] “Test methods for the measurement of the operational characteristics of brushes for electrical machines”. Figure 2 is a schematic diagram of the testing device, in which the wear ring with a diameter of 144 mm was made of copper alloy (C61400, ASTM). Brush samples with dimensions of 16 mm × 8 mm × 5 mm, as shown in Figure 3, were installed in the notches of fixtures A and B. The experiment was conducted with a loading of 4.9 N and a sliding speed of 18 m/s; the test duration was 8 h for each sample. Both mechanical friction and electric friction were used to study the wear resistance of samples under non-conductive and conductive conditions with 8A direct current. In terms of equipment error, the error range for length was ±0.05 mm and for force it was ±0.05 N.

## 3. Results and Discussion

### 3.1. Physical Properties of the Composites

**Figure 4:**
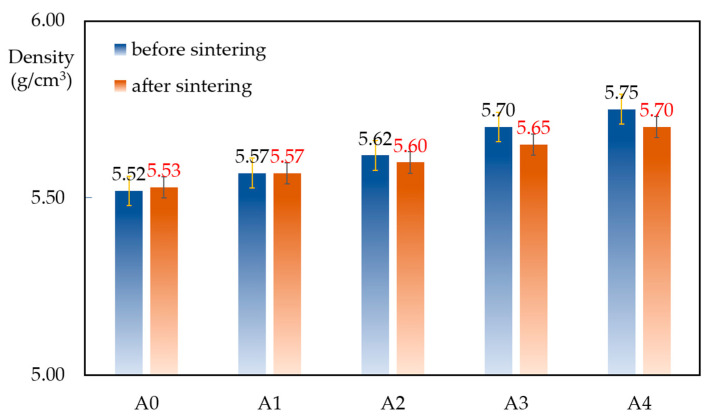
Density of the samples before and after sintering.

Figure 4 shows the density of samples before and after sintering. It is clear to see that, with an increasing content of ZrB_2_, the density of the samples improved significantly, as the density of ZrB_2_ is almost three times as high as that of graphite. However, the density of the composites decreased after sintering, especially for samples with a higher content of ZrB_2_. Additionally, a slight volumetric expansion may have occurred, which was closely related to the limitations of hot-pressing sintering, as mentioned in Zhang et al. [22].

**Figure 5:**
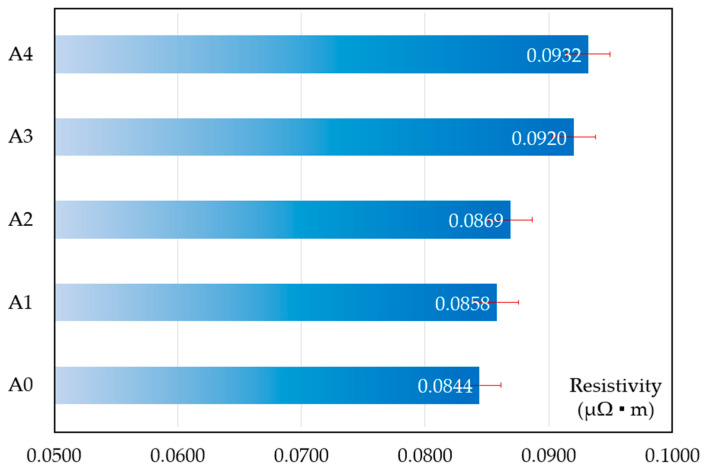
Resistivity of the samples after sintering.

Figure 5 displays the resistivity of samples with increasing content of ZrB_2_ after sintering. It is obvious to see that the resistivity increased. In accordance with previous research, the electron-scattering effect of nano-ceramic particles increased with an increasing content of ZrB_2_, which improved the resistivity of the composite [23]. Moreover, the strengthening effect of the thermal expansion mismatch after sintering contributes to a reduction in electrical conductivity as well [24].

### 3.2. Mechanical Properties of the Composites

**Figure 6:**
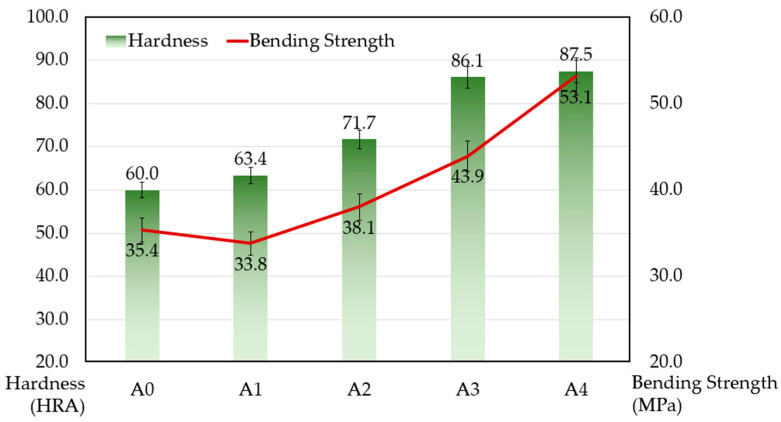
Hardness and bending strength of the samples after sintering.

To study the mechanical properties of the composites, the hardness and bending strength of the samples were measured, and the results are shown in Figure 6. It is clear to see that, as the content of ZrB_2_ increases, both the hardness and the bending strength of the samples display an obvious upward trend. Compared with the samples without ZrB_2_, samples with the addition of 4% ZrB_2_ achieved a hardness of 87.5 HR, improving by 45.8%, and bending strength reached 53.1 MPa, increasing by nearly 50.0%.

Basically, ZrB_2_ particles have higher hardness than graphite. The nanoscale particles distributed in copper–graphite grains could improve their resistance to the migration of dislocations and sub-boundaries. These ZrB_2_ nanoparticles, which disperse into the copper matrix, transform the dislocation pile-up mechanism into a dislocation pinning network mechanism. In addition, due to the difference in the thermal expansion coefficient between ZrB_2_ and Cu, large internal stress is generated during the sintering process, which leads to lattice distortion and increases dislocation density; as a result, the bending strength and hardness of the composites is improved [25].

**Figure 7:**
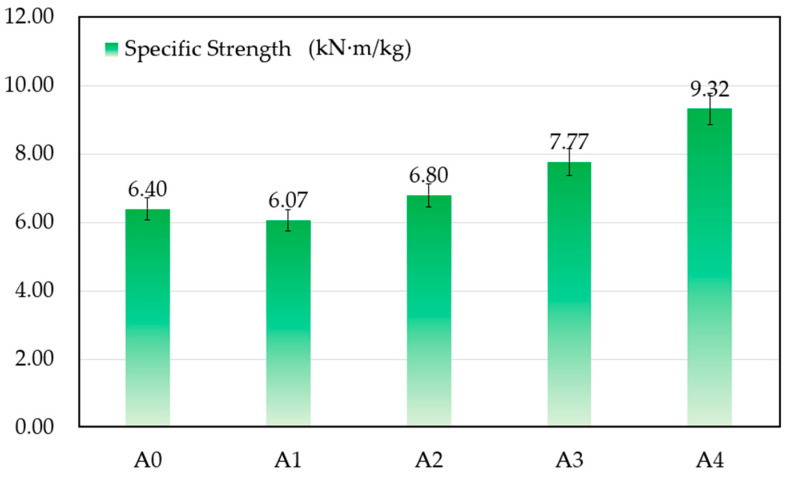
Specific strength of the samples after sintering.

As lightweighting has become an important factor in component and product design and production, the density of the samples has been taken into consideration to calculate their specific strength. Figure 7 shows the results of each sample group. With a 4% addition of ZrB_2_, the specific strength of sample A4 reached 9.32 kN·m/kg, which is about 20% higher than that of sample A3 and 46% higher than that of samples without ZrB_2_. The results provide a vital reference for designing and preparing lightweight structural materials for practical applications.

**Figure 8:**
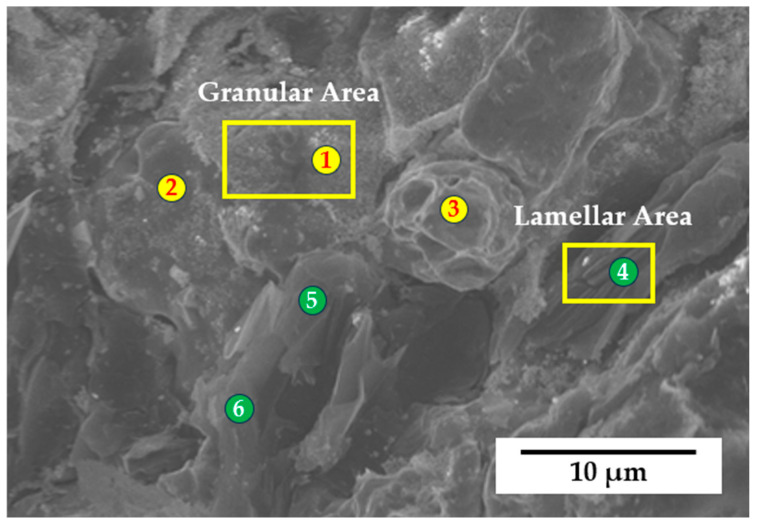
Typical fracture morphology of sample A4 after bending test.

SEM was applied for the section analysis of samples after the bending test. Figure 8 shows a typical fracture morphology of the composites. ZrB_2_ nanoparticles are non-uniformly distributed in the copper matrix. It is obvious that there are two different areas in Figure 8, namely, a granular area and lamellar area. EDS was used to study the chemical composition of these areas. The spectrum and results of the EDS test are shown in Table 2. It is clear to identify that the lamellar area is graphite-dominant and the granular area is zirconium–copper-composite-dominant in nanoscale. Given that graphite has a lower shear strength, most cracks occurred in the lamellar area. According to a previous study [26], distributed ZrB_2_ particles tend to aggregate into agglomerations and this trend is more pronounced with the increasing content of ZrB_2_ in the matrix.

### 3.3. Wear Resistance of the Composites

**Figure 9:**
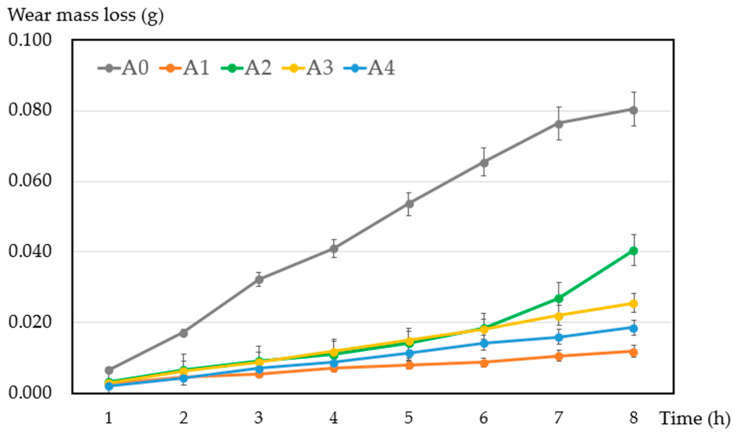
Wear mass loss of the samples after mechanical friction.

The wear mass loss of the samples after mechanical friction was measured and is displayed in Figure 9. The wear mass loss of each sample was measured three times to calculate the average value. With the addition of ZrB_2_, the wear mass loss of samples was significantly reduced compared with sample A0. ZrB_2_ nanoparticles have higher hardness and were dispersed in the copper–graphite composites, which improved their strength. Sample A1 (with 1% ZrB_2_) shows the best wear resistance with a minimal average wear mass loss of 0.012 g, which is a reduction of about 85.2% compared with sample A0 without the addition of ZrB_2_. However, the wear mass loss of samples A2, A3, and A4 reveals a decreasing trend with an increasing content of ZrB_2_. On the one hand, the effect of dispersion strengthening is more obvious with an increasing content of ZrB_2_ nanoparticles; on the other hand, ZrB_2_ nanoparticles bear a certain load that prevents direct contact against the friction pair.

**Figure 10:**
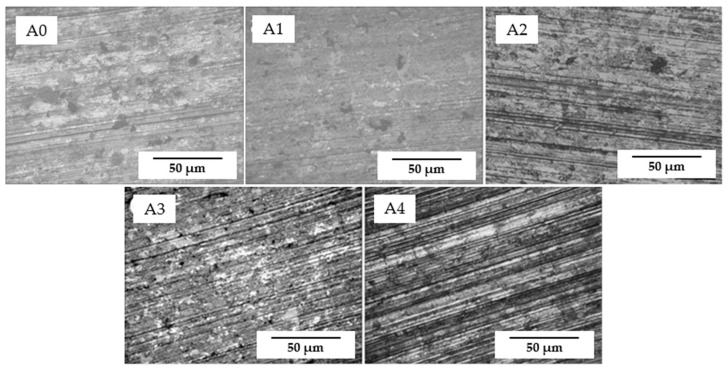
Typical surface morphology of samples after mechanical friction.

Similar results can be found from the surface morphologies of the samples as well. As shown in Figure 10, it is obvious to see that sample A1 (with 1% ZrB_2_) has the best surface state, with little deformation and very shallow grooves, which indicates the best wear resistance and is in accordance with the result of wear mass loss. A protective membrane which played the role of a lubrication film was formed during the friction process, and its content was detected as a composite of ZrB_2_ and graphite. Compared with sample A0, the surface of samples with the addition of ZrB_2_ contains less spalling of graphite and the wear surface is much smoother because the dispersion of ZrB_2_ enhanced the bonding of the graphite–copper composite, leading to higher hardness, as displayed in Figure 6. However, with increases in the content of ZrB_2_, the lubrication film disappeared, and the wear grooves became significantly deeper, which is typical abrasive wear.

**Figure 11:**
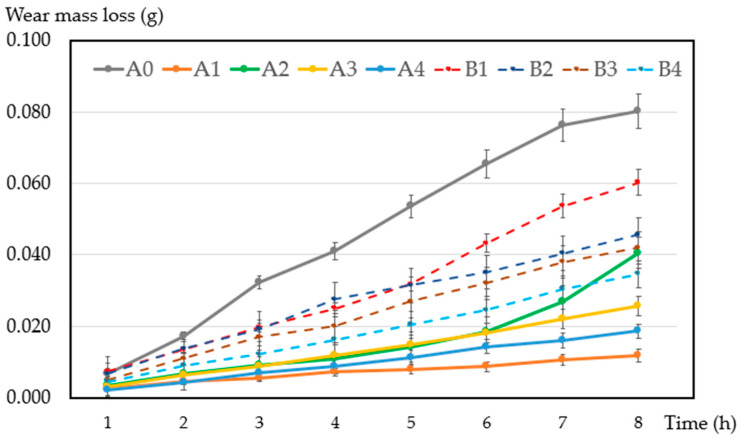
Wear mass loss of the samples after conductive friction.

Figure 11 shows the results of wear mass loss of samples under both non-conductive friction and conductive friction. Compared with samples A1, A2, A3, and A4 under non-conductive friction, samples B1, B2, B3, and B4, with the same chemical composition, under conductive friction showed larger wear mass loss during the whole experiment duration. With an increasing content of ZrB_2_, the samples exhibited less wear mass loss. Sample B4 with a 4% addition of ZrB_2_ shows the best wear resistance.

**Figure 12:**
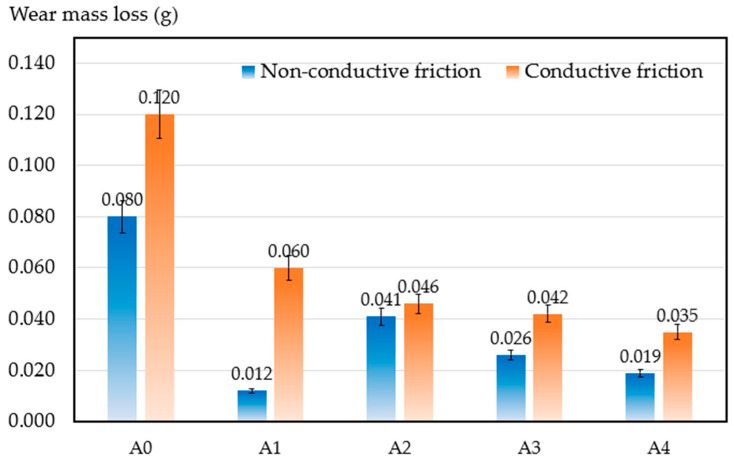
Wear mass loss of the samples after 8 h.

After 8 hours’ friction, the mass of each sample was measured to calculate wear mass loss. Figure 12 shows the wear mass loss of each sample under both non-conductive friction and conductive friction. Under conductive friction, the wear resistance of samples increased with increasing content of ZrB_2_. The sample with 4% ZrB_2_ displayed a nearly 50% reduction in wear mass loss compared with the sample with 1% ZrB_2_.

Generally, samples exhibit lower wear resistance under conductive friction than under non-conductive condition. The average wear mass loss of samples under conductive friction is 87.6% higher than that under non-conductive friction as additional joule heat is generated, accompanied by electrical arc ablation on the surface. Sample surfaces were partially oxidized at higher temperatures and some brittle cracks were produced. In addition, the wear mechanism was changed from delamination-dominant wear to slight-delamination wear with abrasive-dominant wear [19].

**Figure 13:**
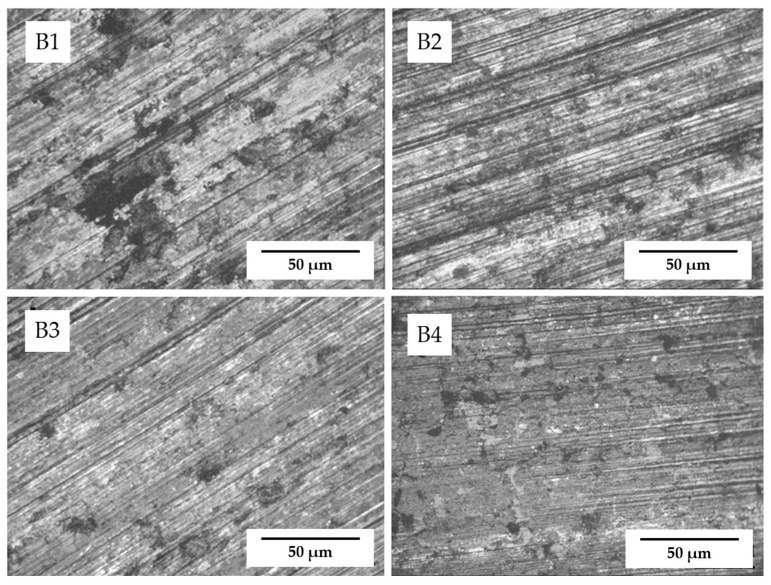
Typical surface morphology of samples after conductive friction.

Figure 13 shows the typical surface morphology of samples after conductive friction. The sample display less spalling of graphite and shallower grooves with increasing content of ZrB_2_. It is worth noting that the lubrication film that occurred on sample A1 was destroyed under conductive friction.

### 3.4. Performance–Cost Analysis

In light of the results above, the overall performance of the composite mainly depends on the content of ZrB_2_ addition. The purchasing price of ZrB_2_ nanoparticles is about 360 USD/kg; on the other hand, copper powder costs 22 USD/kg and graphite powder costs only 2 USD/kg. ZrB_2_, as raw material, takes the highest proportion of the total production cost. Table 3 shows the cost calculation of the samples, which helps us to evaluate their potential industrial application from an economic perspective.

**Figure 14:**
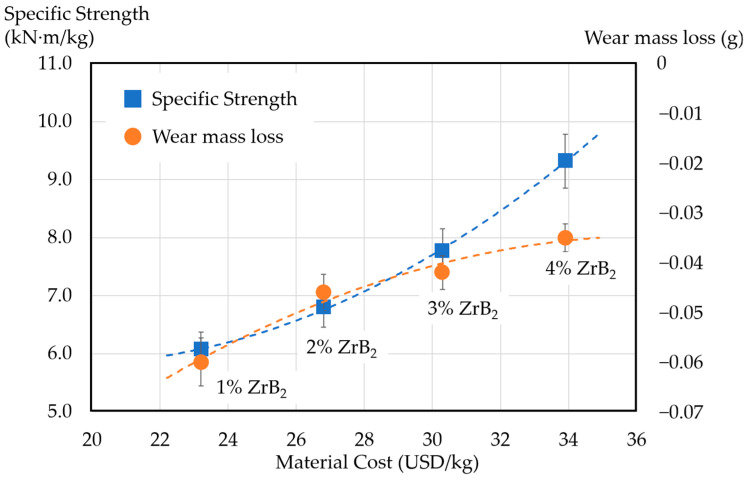
Correlation between sample performance (specific strength and wear mass loss) and material cost.

Figure 14 shows the correlation between sample performance and material cost. The horizontal axis represents the material cost, the vertical axis on the left represents the specific strength of the samples, and the right vertical axis represents the wear mass loss of the samples during conductive friction. With increasing specific strength and wear resistance, the material cost increases as well. Based on the current results, the trend of the specific strength curve shows a higher gradient after the addition of 2% ZrB_2_; however, the wear mass loss curve displays a lower gradient instead.

## 4. Conclusions

This study focused on the improvement of the wear resistance of ZrB_2_–copper–graphite composites for electric brush materials. The effect of ZrB_2_ content on the composites’ mechanical and wear properties was studied. The following conclusions can be drawn:

(1) With an increasing content of ZrB_2_, the density, resistivity, hardness, bending strength, and wear resistance of the composites were enhanced. Compared with samples without ZrB_2_, samples with a 4% addition of ZrB_2_ achieved a hardness of 87.5 HRA, which is an improvement of 45.8%, and bending strength reached 53.1 MPa, which is an increase by nearly 50.0%;
(2) Under non-conductive friction, the composite with 1% addition of ZrB_2_ displays the best wear resistance due to the formation of a lubrication film composed of ZrO_2_ and graphite. Wear mass loss during the friction test was reduced by 85.2% compared with samples without ZrB_2_;
(3) Under conductive friction, all samples showed lower wear resistance. The average wear mass loss of the samples was 87.6% higher than that under non-conductive conditions. The composite with a 4% addition of ZrB_2_ displayed the best wear resistance;
(4) The performance–cost analysis shows that improvements in specific strength and wear resistance are accompanied by increasing material costs.

This study provides a method to improve the performance of electric brushes by adding ZrB_2_ into the current copper–graphite brush materials. However, the optimal composition ratio and sintering parameters should still be investigated further.

## Figures and Tables

**Table 1:** Composition of ZrB_2_–copper–graphite composites (wt.%).

Sample Number	Copper	Graphite	ZrB_2_
A0	88	12	0
A1	88	11	1
A2	88	10	2
A3	88	9	3
A4	88	8	4

**Table 2:** EDS results of spectrum scan for the positions marked in Figure 8.

Area	Position	Cu (wt.%)	Zr (wt.%)	O (wt.%)	C (wt.%)	B (wt.%)
Granular	1	70.7	10.2	4.3	12.5	2.3
	2	81.2	8.9	2.1	6.7	1.1
	3	84.5	7.6	4.4	1.9	1.6
Lamellar	4	17.2	9.7	1.2	68.9	3.0
	5	16.1	5.8	6.7	70.5	0.9
	6	17.8	7.2	6.3	67.4	1.3

**Table 3:** Cost calculation of the samples based on composition proportions.

Sample Number	Copper	Graphite	ZrB_2_	Cost USD/kg
A0	88%	12%	0	19.6
A1	88%	11%	1%	23.2
A2	88%	10%	2%	26.8
A3	88%	9%	3%	30.3
A4	88%	8%	4%	33.9

## Data Availability

Data are contained within the article.
